# Microalgal Biodiesel Production: Realizing the Sustainability Index

**DOI:** 10.3389/fbioe.2021.620777

**Published:** 2021-05-28

**Authors:** Reeza Patnaik, Nirupama Mallick

**Affiliations:** Department of Agricultural and Food Engineering, Indian Institute of Technology Kharagpur, Kharagpur, India

**Keywords:** microalgae, biodiesel, sustainability, economic, environmental, social

## Abstract

Search for new and renewable sources of energy has made research reach the tiny little tots, microalgae for the production of biodiesel. But despite years of research on the topic, a definitive statement, declaring microalgae as an economically, environmentally, and socially sustainable resource is yet to be seen or heard of. With technological and scientific glitches being blamed for this delay in the progress of the production system, an assessment of the sustainability indices achieved so far by the microalgal biodiesel is important to be done so as to direct future research efforts in a more coordinated manner to achieve the sustainability mark. This article provides a review of the current economic, environmental, and social status of microalgal biodiesel and the strategies adopted to achieve them, with suggestions to address the challenges faced by the microalgal biodiesel production system.

## Introduction

Living in an age where life revolves around energy in all forms, a crisis of sustainability is indeed indispensable. With the continued consumption of fossil fuels by the expanding populations, maintaining economic, environmental and social sustainability is a difficult proposition. Hence, strong abatement practices and policies to encourage research on renewable energy resources are being developed. It is in this context that energy in the form of biofuels is being produced from renewable resources of plant origin. Although various other alternatives like geothermal, wind and solar energy are being surveyed, bioenergy is looked at as a strong resource of energy in the coming years.

In such a scenario, the presence of objectionable facts such as issues of food security and energy balance in the first- and second-generation biofuels and the desire for new, sustainable energy resources has brought into limelight, a garden pond nuisance, microalgae, as a promising renewable fuel feedstock. Reports of its high oil yields, dramatic GHG savings, faster growth rate, more harvesting cycles and higher carbon fixation rates, all devoid of any negative effects on farming are reasons of its sudden popularity (Balat and Balat, 2010).

Research on microalgae as a source of energy were extensively carried out in the 1970s, in the United States, but shortage of adequate funding and shift of focus to other feedstocks and technologies gradually brought an end to the research program (Demirbas, 2011). However, with the spurt of concerns today, regarding climate change, food vs. fuel feud, land use change, etc., resulting due to the use of first- and second-generation biofuel feedstocks, the need for search of alternative energy sources has aroused and reawakened interest in microalgae. Although microalgae possess several advantages as compared to first- and second-generation biodiesel feedstocks and are being experimented on different aspects worldwide, sustainable microalgal biodiesel production appears to be a difficult target to reach with regard to its economic, environmental and social positioning. This review extends its scope to identifying the sustainability indices achieved through microalgal biodiesel production and addressing the knowledge gaps in this area for focused research and innovations.

## Sustainable Biofuels

### Definition

**FIGURE 1:**
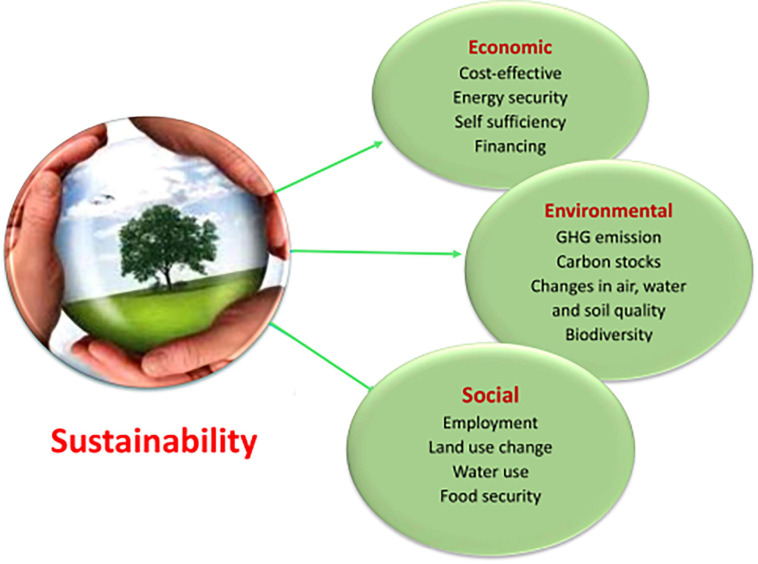
Economic, environmental and social aspects of sustainable biofuels.

The term ‘sustainability’ has been rightfully defined by the World Commission on Environment and Development as “the development that satisfies the needs of the present generations without compromising the ability of the future generations to meet their own needs.” Sustainable development comprehends economic, social, and ecological standpoints of conservation and change (Figure 1) (UNCED, 1992).

Despite the widespread use of the term, ‘sustainability,’ human beings fail to cater to the basic requirements for a sustainable society which is clearly reflected through their activities of environmental degradation, overconsumption, population growth and their quest for indefinite economic growth in a closed system.

### Parameters to Be Considered for Sustainable Biofuel Production

For achieving economic sustainability, low-cost production strategies with greater output to input ratio is imperative besides being available at affordable market rates. At times, the need to maximize returns from investments overlooks the environmental considerations giving rise to negative implications. Additionally, the demand for economic gains affects food production and availability, creating adverse impacts on the society. Hence, to balance between economic and environmental sustainability, higher productivity must be targeted.

Environmental sustainability can be assessed through use of environment friendly, renewable sources of energy along with use of chemicals and machines during the production process with minimum negative environmental impact. These assessments are done with the help of some indicators which can either be global (GHG emissions, renewable energy) or regional (water management, soil and resource depletion, local pollution, etc.). Moreover, the government and private led directives, schemes and initiatives for spreading awareness and activity, also contribute toward environmental sustainability in a major way (Afgan, 2008).

Social sustainability can be ascertained through implementations of certification schemes, scorecards and regulations for mitigating the negative impacts such as child labor, minimum wage, compensation for lost land and resources etc (Haye and Hardtke, 2009). However, evidences of implementation of these measures in reality, has been very limited, suggesting lower degrees of interest or awareness for establishing social sustainability. Low social and political participation and contrasting social norms have been few of the many reasons for this debacle. Hence a participation of society and resources for a collaborative effort toward social and economic development and sustainability should be planned.

The basic criteria and indicators for production of sustainable biofuels have been clearly stated by Silva Lora et al. (2011) (Table 1). The report also mentions that for assessing the sustainability of biofuels, parameters like life cycle impact assessment, quantification of substituted fossil energy, energy allocated for co-product development and changes in soil utilization should be importantly considered.

**TABLE 1:** Criteria and sustainability indicators for sustainable biofuel production (Silva Lora et al., 2011).

Criteria	Sustainability indicators
(i) Should be carbon neutral in terms of GHG emissions.	(i) Economic indicators (cost of production).
(ii) Should have no negative water footprint and land use change problems.	(ii) Output/input ratio (net energy analysis).
(iii) Should not challenge food security.	(iii) Substituted fossil fuel per hectare.
(iv) Should be economically affordable by the society.	(iv) Avoided GHG emissions (CO_2_ savings).
(v) Should not disturb the biodiversity.	(v) Environmental impacts evaluation using impact categories indicators.
	(vi) Carbon emissions due to land use changes.
	(vii) Renewability indicators (energy accounting).

## Microalgal Biodiesel Production: The Sustainability Check

Algae biodiesel industry is starting to take off. Algae projects see an emerging trend in the production of algal-based *drop in* fuels and various high-value products. In a bid to realize the sustainability index, extensive research efforts are being carried out by researchers, academicians and industrialists worldwide to improve the economic and environmental benefits from algal biodiesel through improvement in upstream and downstream processes. Despite such widespread research activities in the field, many questions still remain unanswered. What exactly is the sustainability index? How far are we from reaching the sustainability mark? Is the same sustainability index applicable to all nations and societies? Although the sustainability index is a concept as vague as, ‘to each his own,’ in this world of expanding problems and populations, to ensure continued efforts in the right direction and at the right pace, a sustainability check of the microalgal biodiesel production system is indispensable.

### Economic Sustainability

Biodiesel production is an energy intensive process. All the processes during biodiesel production, starting from procurement of raw materials to processing, manufacturing, storing and marketing, contribute toward the product’s economic feasibility. The decision to use a particular biodiesel initially depends on its cost competence. So research efforts to bring down the cost of microalgal biodiesel to comparable rates with conventional petroleum diesel are being focused on. Hence, for ensuring cost effectiveness of the microalgal biodiesel, few important strategies are generally followed, such as (i) increasing the amount of energy captured from the atmosphere, (ii) increasing the amount of energy harvested from the microalgal biomass, (iii) increasing the biomass yield of the resource, (iv) increasing the number of co-products produced, (v) decreasing the energy input during downstream processes, and (vi) increasing the ability of the product to be stored for a longer period of time.

Over the past few years, remarkable advancement has been achieved in the microalgal biodiesel production systems, with respect to technological and economic development. While way back in 2010–2011, the microalgal biodiesel was produced at more than $100/GGE (Gallons of Gasoline equivalent) in a paddle- wheel driven microalgal pond cultivation system (National Research Council [NRC], 2012), over the years, through technological advancement, cost of algal biodiesel production has been lowered to $7.50/GGE (National biodiesel board [NBB], 2009) is further estimated to come down to $3.00/GGE by 2030 (Office of Energy Efficiency and Renewable Energy [EERE], 2017). This cost cut can be attributed to modifications in cultivation, strain selection, harvesting and extraction technologies and co-product development, all of which determine the final cost of the product, i.e., biodiesel. Various organizations and companies have proposed different cost reduction strategies. Few examples include the use of jet mixer technology for direct extraction of lipids from wet algal biomass by researchers from University of Utah (Mohanty, 2019), use of patented harvesting and algae oil extraction systems by *Missing Link Technology* and *Algae Venture Systems* (Lane, 2014), and use of patented quantum fracture technology for efficient and innovative single step oil extraction from microalgae by Origin Oil Co., (Eckelberry and Eckelberry, 2008). Interestingly, this invention by Origin Oil Co., claims to reduce microalgal biodiesel cost to $2.00/GGE. In addition to inventions in the production and conversion process, various companies like Sapphire Energy, Muradel, Solazyme, Algae.Tec, Cellana and Neste Oil, BioProcess Algae and Algenol are setting up large-scale production units for attaining commercial feasibility of algal oil (European Technology and Innovation Platform Bioenergy [ETIP], 2014). Through co-ordinated research and development activities, microalgal biodiesel production is gradually moving from economic uncertainity to economic feasibility. Specifics of few selected strategies adopted for reducing the cost of microalgal biodiesel production have been listed in Table 2.

**TABLE 2:** Strategies adopted for reducing the cost of microalgal biodiesel production.

Sl No.	Estimated cost of microalgal biodiesel ($/gallons of gasoline equivalent)	Strategy adopted	References
(1)	$7.50	Use of a newly discovered microalgal strain, *Chlorella* sp. DOE1412, with a robust ability to accumulate high quantity of lipid under variety of conditions when grown in a self-designed open pond cultivation system, the Aquaculture Raceway Integrated Design (ARID) with an integrated temperature control mechanism, and harvested through electrocoagulation (EC), a low-energy harvesting method for subsequent oil extraction and upgradation to biodiesel without using any solvents in a hydrothermal liquefaction chamber.	National Alliance for Advanced Biofuels and Bioproducts [NAABB], 2014
(2)	$2.68 $1.58 $3.67 $2.11	Simultaneous lipid extraction and transesterification in a mixer containing methanol and sodium hydroxide through ultrasonication of the harvested microalgal biomass with an annual average productivity of 30 g/m^2^/day using CO_2_ from flue gas 60 g/m^2^/day using CO_2_ from flue gas 30 g/m^2^/day using pure CO_2_ 60 g/m^2^/day using pure CO_2_.	Nagarajan et al., 2013
(3)	$4.35	Use of a high lipid containing (41% dcw) microalgal strain with an annual average productivity of 30 g/m^2^/day processed through hydrothermal liquefaction technique and purified for biodiesel production while simultaneously utilizing the spent biomass for production of other value-added products such as bioethanol and methane. Re-circulation and re-use of water and solvents through the biorefinery system has also been applied.	Davis et al., 2011

Although microalgal biodiesel production is a topic being researched worldwide, reports on detailed cost analysis of the final product (as an effect of the entire production system), is limited, nevertheless production models with cost reduction calculations anticipating a competitive market for microalgal biodiesel, substitute the limitation (Richardson et al., 2010; Harun et al., 2011). Microalgal biodiesel is gradually moving toward being more cost effective but complete economic parity with petroleum diesel is yet to be realized. With diesel currently costing $3.08/gal (Diesel Prices, 2021) on an average throughout the world, to make it comparable with algal diesel an equivalent market price is inevitable. For this to be achieved, algal biomass yields (given that all integrated systems based on algal biomass processing are constrained by high cultivation variability) will have to be increased approximately from 12 to >30 gdw/m^2^/day on a sustained basis, the energy-return-on-investment (EROI) for harvesting algae from ponds ideally would need to be >20, i.e., no more than 5% of the energy content of the algae should be spent during harvesting and the lipid extraction and conversion efficiency to biodiesel should be improved so as to ensure minimum expenses in the defined process (Stephens et al., 2010; Olivieri et al., 2013; Barry et al., 2015; Barsanti and Gualtieri, 2018). Additionally, further lessening of microalgal biodiesel prices can be accomplished by focusing on maximizing lipid content in high biomass yielding microalgal strains and valorization of the algal biomass, as it results in more substantial cost reduction (Bellou et al., 2014; Zhu, 2015). Photobioreactors are also known to be very effective for producing high biomass and lipid productivities, but given the construction and operation challenges such as overheating, fouling, improper gas exchange etc., this option appears less sustainable for commercial use. A study by Veeramuthu and Ngamcharusrivichai (2020) demonstrated algal biodiesel cost to be $20.53 and $9.84 per gallon using a PBR and open raceway pond cultivation method, respectively. Reports in support of this claim have also been published by various other researchers and companies like Weissman et al. (1988), Craggs et al. (2011), Efroymson et al. (2020), Ganesan et al. (2020), Jo et al. (2020) including National renewable energy laboratory (Davis, 2017) and the Solix (Kanellos, 2009). In Table 3 below, different strategies being followed in the last 5 years over and above the ones mentioned in Table 2 above, with an anticipation to reduce the final selling price of microalgal biodiesel, are highlighted.

**TABLE 3:** Research efforts in the last 5 years with an anticipation to reduce the final selling price of microalgal biodiesel.

Sl. no.	Targeted step	Adopted strategy	References
(1)	Strain improvement	Fluorescence-activated cell sorting to analyze single-cell fluorescence and sort cells with high fucoxanthin and lipid productivities.	Gao et al., 2021
(2)	Strain improvement	Post-treatment processing using H_2_SO_4_ + Ca (OH)_2_ for enhanced ethanol production from algae.	Seon et al., 2020 Südfeld et al., 2021
(3)	Strain improvement	Use of coral inspired 3D materials for higher biofuel production by increasing the photon resident time for enhanced light absorption by algal cells.	Wangpraseurt et al., 2020
(4)	Strain improvement	Strain improvement through high-throughput screening platforms i.a. involving single-cell methodologies such as fluorescence-activated cell sorting (FACS) for the identification and isolation of better-performing strains by combining qualitative staining of lipid bodies using the fluorophoric dye BODIPY with FACS methodology.	
(5)	Strain improvement	Use of broad range and wide variety of carbon sources for enhancing growth and lipid accumulation in algae.	Patnaik and Mallick, 2019
(6)	Strain improvement	Researchers at Tokyo Institute of Technology have identified an enzyme belonging to the glycerol-3-phosphate acyltransferase (GPAT) family as a promising target for increasing biofuel production from the red alga *Cyanidioschyzon merolae*.	Fukuda et al., 2018
(7)	Strain improvement	Researchers at Los Alamos National Laboratory, with colleagues at NREL and the University of Georgia report that a freshwater production strain of microalgae, *Auxenochlorella protothecoides* UTEX 25, is capable of directly degrading and utilizing non-food plant substrates, such as switchgrass, for cell growth. In addition, the use of plant substrates increases lipids production.	Vogler et al., 2018
(8)	Strain improvement	Rapid screening of high lipid accumulating microalgal strains through droplet microfluidics based screening platform.	Kim et al., 2017
(9)	Strain improvement	Doubling of lipid content while sustaining growth using CRISPR-Cas 9 for modulating a transcriptome regulator in *Nannochloropsis gaditana*.	Ajjawi et al., 2017
(10)	Strain improvement	Discovery of an algal photoenzyme that converts algal fatty acids to alkanes and alkenes under low-light driven conditions.	Sorigué et al., 2017
(11)	Microalgal cultivation and valorization	Use of iron oxide nanoparticles for improved growth and biogas production in algae.	Rana et al., 2020
(12)	Microalgal cultivation	Use of tannery wastewater for growth and biofuel production from green microalgae through bioremediation.	Nagi et al., 2020
(13)	Microalgal cultivation	Outdoor open pond batch production of green microalga *Botryococcus braunii* for high hydrocarbon production using different salinity concentrations.	Ruangsomboon et al., 2020
(14)	Microalgal cultivation	Use of iron and magnesium addition for improving population dynamics and high value product formation in microalgae grown in anaerobic liquid digestate.	Ermis et al., 2020
(15)	Microalgal cultivation	A simplistic approach of algal biofuels production from wastewater using a Hybrid Anaerobic Baffled Reactor and Photobioreactor (HABR-PBR) System.	Khalekuzzaman et al., 2019
(16)	Microalgal cultivation	Use of 40,000L closed raceway ponds for algal growth and lipid accumulation under biphasic nitrogen starved conditions.	Bagchi et al., 2019
(17)	Microalgal cultivation	Biomimetic light dilution using side-emitting optical fiber for enhancing the productivity of microalgae reactors. This technique enables homogeneous illumination of large reactor volumes with high optical density eventually increasing the rate of reproduction by 93%.	Wondraczek et al., 2019
(18)	Microalgal cultivation	Multi-bandgap Solar Energy Conversion via Combination of Microalgal Photosynthesis and Spectrally Selective Photovoltaic Cell for higher biomass production.	Cho et al., 2019
(19)	Microalgal cultivation and product extraction	Discovery of a new mechanical algal milking technique for extracellular production of polysaccharides and phycobilliproteins.	Uchida et al., 2020
(20)	Product extraction	Pulsed Electric Fields-Assisted Extraction of Valuable Compounds From *Arthrospira Platensis*.	Carullo et al., 2020
(21)	Product extraction	Electroporation as a Solvent-Free Green Technique for Non-Destructive Extraction of Proteins and Lipids From *Chlorella vulgaris*.	Eleršek et al., 2020
(22)	Microalgal harvesting	Effective harvesting of *Nannochloropsis* microalgae using mushroom chitosan	Chua et al., 2020
(23)	Microalgal harvesting and valorization	Induction of flocculation and photobiological hydrogen production under anaerobic conditions using an engineered chemoenzymatic cascade system.	Chen et al., 2020
(24)	Microalgal cultivation and harvesting	Use of a Tris-Acetate-Phosphate-Pluronic (TAPP) medium that undergoes a thermoreversible sol-gel transition to efficiently culture and harvest microalgae clusters without affecting the productivity as compared to that in traditional culture in a well-mixed suspension.	Estime et al., 2017
(25)	Microalgal harvesting	Use of pine bark, a natural substrate for immobilization of microalgae grown in wastewater for easy and cost-effective separation of algal cells.	Garbowski et al., 2020
(26)	Microalgal harvesting	Use of cellulose nanofibrils for cost-effective microalgal harvesting through encapsulation of microalgal cells by nanofibrous structure formation.	Yu et al., 2016
(27)	Microalgal valorization	To develop a thin-layer artificial biofilm technology for sustainable and long-termethylene photoproduction, where recombinant *Synechocystis* sp. PCC 6803 cells holding ethylene forming enzyme (Efe) from *Pseudomonas syringae* are entrapped within the natural polymer matrix, thus forming the thin-layer biocatalytic structure.	Vajravel et al., 2020
(28)	Microalgal valorization	*Chlorella vulgaris* extract as a serum replacement that enhances mammalian cell growth and protein expression.	Ng et al., 2020
(29)	Microalgal valorization	Researchers were able to increase hydrogen production by combining unicellular green alga called *Chlamydomonas reinhardtii* with *Escherichia coli* bacteria. The teamwork of the algae and bacteria resulted in 60% more hydrogen production than they are able to produce if algae and bacteria work separately.	Fakhimi et al., 2019
(30)	Microalgal valorization	Use of algal protein from the de-oiled biomass as a replacement of the commercially available fish meal under an algal refinery approach.	Patnaik et al., 2019
(31)	Microalgal valorization	Microalgal Protein Extraction From *Chlorella vulgaris* FSP-E Using Triphasic Partitioning Technique With Sonication.	Chia et al., 2019
(32)	Microalgal valorization	Mild Fractionation of Hydrophilic and Hydrophobic Components From *Neochloris oleoabundans* Using Ionic Liquids.	Desai et al., 2019
(33)	Microalgal valorization	Synthesis of benzene, an elementary petrochemical, along with other hydrocarbons.	Pingen et al., 2018
(34)	Downstream processing	A synthetic protocol to the fixation of carbon dioxide by converting it directly into aviation jet fuel using novel, inexpensive iron-based catalysts.	Yao et al., 2020
(35)	Downstream processing	The use of jet mixer technology for direct extraction of lipids from wet algal biomass by researchers from University of Utah.	Mohanty, 2019
(36)	Downstream processing	Low-temperature catalyst based Hydrothermal liquefaction of harmful Macroalgal blooms, and aqueous phase nutrient recycling by microalgae.	Kumar et al., 2019
(37)	Downstream processing	Bleaching, deoxygenation and hydroisomerization of crude extracted algal lipids to renewable diesel.	Kruger et al., 2017
(38)	Downstream processing	Establishment of axenic cultures of armored and unarmored marine dinoflagellate species using density separation, antibacterial treatments and stepwise dilution selection.	Lee et al., 2021
(39)	Technique and technology advancement	A simple and non-destructive method for chlorophyll quantification of *Chlamydomonas* cultures using digital image analysis for easy and fast assessment of growth.	Wood et al., 2020
(40)	Technique and technology advancement	Metabolomics as a tool for understanding the molecular basis for these metabolic and physiological changes, and for early detection of stress in freshwater alga *Poterioochromonas malhamensis* exposed to silver nanoparticles.	Liu et al., 2020
(41)	Technique and technology advancement	Development of a pVEC peptide-based ribonucleoprotein (RNP) delivery system for genome editing using CRISPR/Cas9 in *Chlamydomonas reinhardtii*.	Kang et al., 2020
(42)	Technique and technology advancement	Development of a species-specific transformation system using the novel endogenous promoter calreticulin from oleaginous microalgae *Ettlia* sp.	Lee et al., 2020

Reduction in algal biodiesel costs through coupling of economic and environmental sustainability, is yet another emerging potential strategy for the future. Use of fossil fuel releases CO_2_ into the atmosphere which is sequestered back by microalgae for growth and product development. This technology called the BECCS has been rated as the most technologically and economically potential solution for mitigating the impact of GHG emissions, by the IPCC (National biodiesel board [NBB], 2009). Additionally, tax credits and/or carbon credit policies provide further cost reductions by incentivising carbon capture for bioenergy production (Sayre, 2010). In the following section, we consider details of the suggested strategy for achieving environmental sustainability while taking care of the cost effectiveness that is consequent to the entire process.

### Environmental Sustainability

The impact that the microalgal biodiesel production process has on the environment during its entire life cycle decides its environmental sustainability. Starting from the choice of the cultivation area to use of nutrients for growth and lipid accumulation enhancement, to use of different energy intensive harvesting techniques followed by extraction of lipids using different extracting solvents and then conversion of the extracted lipid to biodiesel, all contribute toward the environmental sustainability of the product. This sustainability index can be verified by the use of certain indicators such as GHG emissions, energy security, water management, soil and resource depletion, local pollution, etc. Tools such as Life Cycle Impact Assessment (LCIA) are used for measuring these indicators. LCAs can highlight areas of concern and focus the future research efforts on aspects of the supply chain that carry the largest environmental burden (US Environmental Protection Agency [EPA], 2010).

Global warming due to increasing concentrations of greenhouse gases in the atmosphere is a daunting environmental challenge in today’s world. Of the different greenhouse gases present, CO_2_ is majorly responsible for this problem. CO_2_ is naturally present in the atmosphere, but activities such as burning of forests, mining and burning coal increase their concentrations to dangerous levels in the atmosphere by converting the carbon stored in the solid state to gaseous state (Sayre, 2010). Microalgae are widely known for being potential sequesters of large amounts of CO_2_ from the atmosphere thus lowering GHG emissions relative to petroleum diesel. Additionally, their ability to recycle the released CO_2_ from the different stages of the microalgal biodiesel process within their own system, categorizes them as an environmentally sustainable resource. Many researchers have reported that algal biodiesel has the ability to reduce the GHG emissions by half (55,400 g of CO_2_ equivalent per million BTU) as compared to what is emitted by low sulfur diesel fuel (101,000 g of CO_2_ equivalent per million BTU) (Brune et al., 2009; Gude et al., 2012). This is further confirmed by the United States Environmental protection agency as per which algal/microalgal biodiesel has the potential to meet the Renewable Fuel Standard requirement 2007 by reducing 50% of GHG emissions as compared to petroleum diesel (Sissine, 2007). With petroleum diesel having GHG emissions about 90 g CO_2_ eq/MJ of fuel, for warranting minimum negative impact on the environment, several strategies have been developed and adopted worldwide in order to reduce CO_2_ emissions, details of which have been listed in Table 4.

**TABLE 4:** GHG emissions by microalgal biodiesel production system and strategies adopted for their reduction.

Sl no.	GHG emissions (g CO_2_eq/MJ of biodiesel)	Strategy adopted	References
(1)	−11.4	In this Well to Wheel (WTW), *Scenedesmus dimorphous* with an annual biomass productivity of 13 g/m^2^/day was cultivated in open raceway ponds fed with fertilizer grade N, P, K and industrial flue gas as carbon source. The entire process chain moved from harvesting of the biomass using bio-flocculation and dissolved air floatation followed by centrifugation to use of hydrothermal liquefaction for further processing to bio-oil. The energy expenses and GHG emissions were balanced by recycling of nutrients present in the aqueous phase from the HTL unit, bypassing the need of drying and the co-product credits of the combustible gases emitted from the hydrothermal system for improving the energetics of the biodiesel production process.	Bennion et al., 2015
(2)	71	In this Well to Wheel (WTW), green microalgae with an annual biomass productivity of 25 g/m^2^/day and lipid content of 25% (dcw) was cultivated in open raceway ponds fed with recirculated growth media from the liquid digestates and biogas as carbon source. The entire process chain moved from harvesting of the biomass using bio-flocculation and dissolved air floatation followed by centrifugation to lipid extraction from wet biomass using *n*-hexane and transesterification using methanol. The energy expenses and GHG emissions were balanced by water and 66% nitrogen and 90% phosphorous recycling and the co-product credits of biogas produced during anaerobic digestion and processed through combined heat and power technique for use on-site during the biodiesel production process.	Yuan et al., 2015
(3)	28.50	In this Well to Wheel (WTW) Life cycle analysis, an algal biomass productivity of 20 g/m^2^/day and lipid content of 30% (dcw) was assumed in open raceway ponds fed with nutrients from a wastewater source. The entire process chain moved from harvesting of the biomass through bio-flocculation and gravity clarifiers to use of solar dryers for drying the harvested biomass for lipid extraction by hexane and transesterification to biodiesel by using methanol. The energy expenses and GHG emissions were balanced by 89% of nutrient and solvent recycling and the co-product credits of glycerine produced during transesterification and biogas generated from anaerobic digesters which were used in providing electricity through the entire production process.	Woertz et al., 2014
(4)	35.2	In this Well to Wake (WTW) Life cycle analysis, algae with an annual biomass productivity of 20 g/m^2^/day and a lipid content of 14% (dcw) was cultivated in open raceway ponds fed with nutrients from a wastewater treatment plant (WWTP). The entire process chain moved from harvesting of the biomass using settling tanks followed by centrifugation to processing in a hydrothermal liquefaction unit for bio-oil production and transportation to a refinery for upgradation of the extracted oil eventually transporting it to the airport for use as jet fuel. The energy expenses and GHG emissions were balanced by nutrient recycling of the aqueous phase of the hydrothermal liquefaction unit, but a major contribution to GHG emission neutralization was brought about by the integration of the hydrothermal liquefaction unit to the algal cultivation and dewatering system in the WWTP instead of integrating it in the refinery along with the upgradation unit thus bypassing the extra energy lost in transporting the extracted oil to the refinery for upgradation.	Fortier et al., 2014
(5)	41	In this Well to Wheel (WTW), green microalgae with an annual biomass productivity of 22 g/m^2^/day and lipid content of 30% (dcw) was cultivated in open raceway ponds fed with fertilizer grade N, P, K and waste flue gas as carbon source. The entire process chain moved from harvesting of the biomass using gravity clarifiers followed by centrifugation to lipid extraction from wet biomass using n-hexane and transesterification using methanol. The energy expenses and GHG emissions were balanced by the co-product credits of biogas and methane generated through anaerobic digestion and hydrothermal gasification, respectively, eventually producing heat and electricity through combined heat and power technique for use during the biodiesel production process.	Azadi et al., 2014
(6)	50	In this Well to Wheel (WTW), *C. vulgaris* with an annual biomass productivity of 23.5 g/m^2/^day and lipid content of 25% (dcw) was cultivated in open raceway ponds fed with fertilizer grade N, P, K and waste flue gas as carbon source. The entire process chain moved from harvesting of the biomass using aluminum sulfate flocculation followed by centrifugation to use of waste heat dryer for drying the wet biomass for lipid extraction using *n*-hexane and transesterification using methanol. The energy expenses and GHG emissions were balanced by water and 75% nutrient recycling and the co-product credits of glycerine produced during transesterification, and heat and electricity produced from the residual de-oiled biomass processed through combined heat and power technique for use during the biodiesel production process.	Zaimes and Khanna, 2013
(7)	−46.92	In this Cradle to Grave (CTG) life cycle analysis, microalgae with an annual biomass productivity of 25 g/m^2^/day and lipid content of 30% (dcw) was cultivated in open raceway ponds fed with nutrients from sea water and industrial flue gas as carbon source. The entire process chain moved from harvesting of the biomass using chemical-hydraulic flocculation with aluminum sulfate and filtration followed by drying within a thermal dryer for lipid extraction using hexane and transesterification to biodiesel using methanol. The energy expenses and GHG emissions were balanced by nutrient and water recycling after lipid extraction and transesterification steps	Pardo-Cárdenas et al., 2013
(8)	−53	In this Well to Pond (WTP) life cycle analysis, microalgae with an annual biomass productivity of 25 g/m^2^/day and lipid content of 25% (dcw) was cultivated in open raceway ponds fed with fertilizer grade N, P, K and waste flue gas as carbon source. The entire process chain moved from harvesting of the biomass using settling and dissolved air floatation followed by centrifugation to processing through hydrothermal liquefaction technique for bio-oil production. The energy expenses and GHG emissions were balanced by nutrient recycling from the hydrothermal liquefaction technique and by production of electricity by passing the waste gaseous elements from the hydrothermal chamber to the combined heat and power unit.	Frank et al., 2013

Negative emissions signify an outlet of CO_2_ from the atmosphere whereas reduced emissions signify a reduced inlet of CO_2_ into the atmosphere. Both have their own respective benefits, but with the OECD Environmental Outlook 2050 at the 2011 United Nations Climate Change Conference, suggesting achieving CO_2_ concentration targets at lower than 450 ppm by the Bioenergy for Carbon Capture and Storage Technology (BECCS), negative emissions should be critically pursued (National biodiesel board [NBB], 2009).

**FIGURE 2:**
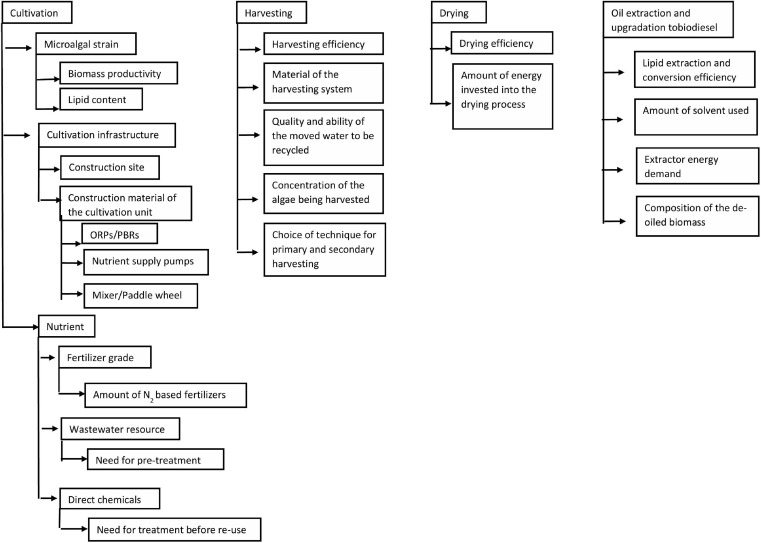
Factors influencing the GHG emissions from the microalgal biodiesel production system.

Microalgae are environmentally sustainable resources emitting green house gases during biodiesel production in quantities lower than that emitted during petroleum diesel production. This can be justified from the Table 4 above, which shows GHG emissions from microalgal biodiesel production systems lesser than 90 g CO_2_ eq/MJ of fuel, recorded for petroleum diesel. But this may not always be true, as microalgal biodiesel production systems in certain cases emit more than 2–10-fold higher greenhouse gases as compared to petroleum diesel (Zaimes and Khanna, 2013). The reason for this variation lies in differences in operational and input parameters of the microalgal biodiesel production process and the interplay between them and the use to which the produced biodiesel is put to. Additionally, the emissions and NER value may also vary from place to place depending on the government regulations and policies and the co-products produced, as highlighted by a research study on corn ethanol by Farrell et al. (2006). On the mention of the operational and input parameters, it is important to note that there are some influencing factors which decide the GHG emission values of the production chain (Figure 2). The factors primarily are cultivation > harvesting > drying > oil extraction/conversion > transport of feedstock > final fuel product, with a decreasing order of importance as regards to their contribution to the final GHG emission figures. Although the figures look promising, yet excessive reliance on few assumptive data sets of selective parameters in some analyses, make way and arouse the need for more elaborate research on the details of the influencing parameters.

Today’s distressing circumstances require that the world emit a total of no more than 1,200 gigatonnes of carbon by the end of this century. That is about 30 years’ worth of carbon emissions at existing levels. But these situations also anticipate absorption of upto 1,000 gigatons of carbon through the above-mentioned merger of bioenergy and carbon capture and storage (CCS), a combination known by the abbreviation BECCS (Azadi et al., 2014). This would then lead to an increase in the total positive emissions (emissions that can be recirculated among the biological system without causing any negative impact on the atmosphere) from 1,200 to 2,200 gigatonnes. Other options such as afforestation, storage of carbon in the soil, and direct air capture of carbon also exist, but are dependent on certain interlinked factors such as land use change and chopping down of trees which transform them from carbon sequestering to carbon releasing strategies. On the other hand, carbon stored in the soil is constantly at the risk of being disturbed. Direct air capture technologies like artificial trees and scrubbing towers are remarkably gee-whiz and show great promise, but are years away from commercialization, currently even more expensive than already very expensive CCS, and we shouldn’t forget that they have a voracious energy appetite themselves. Other possibilities such as the geoengineering techniques of ocean fertilization or enhanced weathering of natural or artificial minerals remain unproven at scale and are already raising hackles amongst some environmentalists. And these are not prominent in any of the considered scenarios (US Environmental Protection Agency [EPA], 2010). As a result, BECCS remains the top bet in the GHG emissions sweepstakes.

Another key metric often considered in microalgal biodiesel analysis is NEB which is defined as the difference between the energy value of the output fuel and the total primary energy consumed in producing the fuel (Zaimes and Khanna, 2013). As such a positive NEB is one important criterion for an environmentally sustainable transportation fuel, because it indicates that more energy is produced than is consumed via the system. EROI and NER are two other energy metrics and represent the ratio of the energy of the final fuel to the direct and indirect primary energy required for its production (Stephenson et al., 2010). Thus, if EROI and NER values are less than unity, then the system has a negative NEB. A variation of EROI known as FER or EROI_*fossil*_ considers only the consumption of primary fossil energy throughout the fuel supply chain and thus measures how much fuel product is generated per unit investment of primary fossil resources. As such EROI_*fossil*_ values provide a surrogate measure for the renewability of the biofuel. Accordingly, EROI_*fossil*_ values more than unity are desirable, because more energy is produced via the biofuel than the fossil energy consumed throughout the supply chain (Vasudevan et al., 2012).

With an energy content of 5–8 kWh/kg (18,000–28,800 kJ/kg dry cell weight), feasibility of microalgal biodiesel production with respect to energy security, can be ascertained if the amount of energy required to produce and process the microalgal biodiesel is found to be lower than the energy contained per dry weight of the alga (Yuan et al., 2015). In current day scenario, petroleum diesel has an EROI_*fossil*_ of 4.64 but the EROI_*fossil*_ of microalgal biodiesel as per published reports is less than unity (Brentner et al., 2011). Various strategies are currently in progress to raise the EROI_*fossil*_ values with some achieving an EROI_*fossil*_ of 1.88 through use of energy efficient harvesting and drying techniques and use of the produced electricity through combined heat and pressure technique for powering the entire production process (Chowdhury et al., 2012) and some others achieving an EROI_*fossil*_ of 2.01 through integration of the microalgal biodiesel system with a wastewater treatment plant (Zaimes and Khanna, 2013). Some researchers are with the belief that microalgal biodiesel can have an EROI_*fossil*_ of 8 (Stephenson et al., 2010), but with current research techniques, for improving the desirability of the microalgal biodiesel, achieving minimum EROI values of 3 is suggested (Marzochella et al., 2010; Vasudevan et al., 2012).

EROI_*fossil*_ and GHG emissions are indirectly proportional to each other with an increase in the value of one parameter bringing about a decrease in the other and vice versa. Hence strategies to reduce the GHG emissions from the microalgal biodiesel production process eventually raise the EROI_*fossil*_ values, thus producing an environmentally sustainable biofuel. EROI is not an absolute indicator of sustainability, but it does help to indicate where a particular source fits in with regional, national and global energy markets. In that context, a competitive EROI for algae biodiesel provides support for a national energy policy that replaces petroleum.

### Social Sustainability

The ability of a product to be sustained for use by the society and for the society, decides its social sustainability. This social dimension of microalgal biodiesel sustainability is decided by its ability to positively impact rural development, poverty reduction and inclusive growth (Elbehri et al., 2013). To judge the impact of the biodiesel production system on the abovementioned indices, factors such as, land ownership rights, local stewardship of common property resources and labor rights are mostly looked into Mohr and Linda (2013). Land being a limited resource, the decision/interest of people holding rights over the land to earn value from it through wealth generation or greening of the environment greatly affects the social sustainability of the microalgal biodiesel. Similarly, stewardship of local common property resources such as community forests, common grounds, threshing grounds, rivers and riverbeds by the co-owners/stewards of the property is another influencing factor as their agreement to the proposal of utilizing the common property resources for bioenergy production at the cost of their dependence on these properties at the time of need is highly essential. The ability of the microalgal biodiesel to generate rural employment and welfare by increasing inflow of capital, fertilizers, infrastructure and technologies to the agricultural/farm sector thus creating new employment opportunities, higher wages and increased self-sufficiency in terms of access to electricity and pumped portable water without causing any negative impact is another unavoidable factor to be considered while deciding the social sustainability of the microalgal biodiesel (Levidow, 2013).

In the market of bioenergy, microalgal biodiesel is like a new born baby waiting to be nurtured and groomed. In such a scenario implementation of social certification schemes, rules, laws or acts for ensuring its social sustainability is too early to be true. Under such circumstances, with the Renewable Fuel Standard, 2007 in United States and Renewable Fuel Quality Directive, 2008 in European Union, mandating a substantial portion of renewable fuel in the transportation sector by 2040, countries all over the world are gearing up with microalgae as a source of biodiesel and encouraging its use by their people through grants to companies equipped for their production (GCC, 2016).

With United States leading the world in microalgal research, majority of research efforts in the field are concentrated here (Gude et al., 2012). Hence the search for the first steps in ensuring social sustainability of microalgal biodiesel can be traced in this nation. The Department of Energy, United States, as part of the nation’s energy strategy had announced ∼$25 million funding to reduce the price of algal biodiesel below $5/GGE by 2019. This funding is believed to support creation of green jobs, innovations, improvement in environment and national energy security. The funding has been partitioned to two phases with the first phase concentrating on valuable co-products development from microalgae besides biodiesel production and the second phase concentrating on carbon capture technologies for improved yields of microalgal biomass (Casey, 2014). Microalgal biodiesel companies with an intention to form strategic partnerships to attract private investments are leveraging co-operative agreements of the Energy Department. For, e.g., Sapphire Energy, an algae based green crude producer and awardee of the DOE funding has signed two commercial contract agreements with Phillips 66 and Tesoro (one being an integrated energy manufacturing and logistics company and the other being an independent refiner and marketer of petroleum products) to upgrade its biodiesel to on-spec diesel which can be used in existing diesel fuel tanks (Liu et al., 2013). Similarly, contract agreements between United States DOE and Hawaii Bioenergy, New Mexico State University and California Polytechnic State University to demonstrate algal biodiesel yields greater than 2,500 gallons per acre with a funding of $ 16.5 million have also been entered into (Office of Energy Efficiency and Renewable Energy [EERE], 2014). In addition to these the United States Government has effectively implemented the Clean Power Plan of the Environmental Protection agency, and has been hailed successful by the Algae Biomass Organization for maximum carbon capture by microalgae, setting federal guidelines for states to reduce carbon emissions by 32% before 2,030 to regulate the concentration of CO_2_, an environmental pollutant, in the atmosphere (Kommers, 2013). Such strategic actions have also been taken by companies in Canada and the European Union and various other parts of the world European biofuels technology platform (EBTP, 2016; NRCC, 2013).

Employment generation by microalgal biodiesel production is a statistic yet to be derived but with the emergence of numerous companies interested in working for biodiesel production from microalgae, employment of laborers in large numbers is expected. Statistics of job creation from biodiesel production in 2011 (first and second-generation biodiesel) shows a support of 39,027 jobs and more than $ 2.1 billion in household income in the United States (national biodiesel board) (National biodiesel board [NBB], 2009). These jobs created by using economic and environmentally sustainable means (biodiesel), are categorized as ‘Green Jobs’ and are more clearly defined by the UNEP as a job in any field of work be it agriculture, manufacturing, R&D etc., that contributes substantially to the preservation and restoration of environmental quality. It is a joint initiative by the UNEP, the ILO, and the ITUC in the year 2007 (United Nations Environmental Programme [UNEP], 2008).

With a rush for jobs by the skilled and educated masses, unemployment among the unskilled rises to alarming levels. In order to balance this difference, programs like Pathways out of Poverty (POP), a national workforce training program by the United States government’s ARRA of 2009 trains individuals living below or near poverty level with skills needed to enter the green job market, focusing primarily on the energy efficiency and renewable energy industries. The training programs focus on teaching basic literacy and job readiness skills in addition to providing supportive assistance with childcare and transportation to overcome barriers to employment (Universidad, 2009).

## Challenges and Avenues for Future Research

Microalgal biodiesel production has been initiated on a pilot scale at various places, but a discussion on their ability to profoundly displace petroleum diesel, has been mostly ignored. In today’s market condition, microalgal biodiesel is more expensive than petroleum diesel as the improved economics of production are inadequate for environmentally sustainable production let aside the oblivion of social sustainability. A retrospection of the different research studies on microalgal biodiesel production system highlights few major challenges in the production of biodiesel from microalgae eventually hindering its commercialization. A few essentials are explicitly addressed.

1. The different stages of microalgal biodiesel production continue to be highly energy intensive impeding attainment of economic and environmental sustainability.
2. A low-cost arrangement for water, nutrients and CO_2_ with minimum negative impact on the environment and microalgal culture quality still appear to be challenging.
3. Maintaining a monoculture inside the raceway ponds continues to be difficult to achieve.
4. Unsuitability of non-native algae to a new ecosystem creates risks of microalgal spills.
5. Scaling up of microalgal culture is a big problem with high degrees of uncertainity about the replication of functional characteristics in the scaled up cultivation system.
6. Huge variation in GHG emissions and EROI data from different research studies question the efficacy of the strategies being adopted.
7. Lack of faster and efficient tools for screening of oleaginous microalgal strains slows down progress in the field.
8. Lack of complete biochemical and molecular profiling of oleaginous microalgae restricts informations and innovations.
9. Lack of detailing of the cultivation and operational parameters used in the microalgal biodiesel production system, hinders complete sustainable development of the production system.
10. Routes for recuperating energy from the microalgal biomass left after oil extraction are required for attaining a net positive energy balance during the production of microalgal biodiesel.
11. Lack of sufficient genetic and metabolic engineering in the field of microalgal biodiesel confines exploration of genes that control the production of lipid in microalgae.
12. Wasted energy from captured photons during photosynthesis is a major challenge in mass algal cultivation.
13. Uncertainities about policy support and competition from other fuels further adds to the plight.

With a focus and determination to defy the pessimistic view of a group of research scientists who claim that microalgal biodiesel can never outcompete petroleum diesel, research organizations, institutions and individuals are working with hastened speed to address the challenges mentioned above. Although fortunately there has been some success in achieving some near-term goals as has been mentioned in the previous sections, there still remains enough work to be done in future, details of which have been mentioned below.

1. With microalgal cultivation requiring huge inputs of nitrogen and phosphorous, recycling of nutrients with special emphasis on the quality and quantity of nutrients being recycled can be focused on.
2. With reports of 100% nutrient recycling raising the cost of microalgal biodiesel by $2/Gal as compared to 0% recycle (Davis et al., 2017), alternative wastewater resources can be tracked and their complete profiling including nutrient and bacterial count can be noted down before being used for cultivation so as to include the pre-treatment costs in the final economics of the produced biodiesel.
3. Co-products reduce the economic and environmental burdens of microalgal biodiesel but life cycle impact assessment studies to understand the type of co-products which when produced provide maximum benefit in attaining sustainability, can be done.
4. Use of paddle-wheels in raceway ponds is where maximum allocation of capital is done. In order to reduce the cost burdens (National biodiesel board [NBB], 2009), alternative, less energy intensive technologies for culture mixing can be explored, notwithstanding the water pumping step which also exerts a substantial energy burden.
5. Combined heat and power treatment of the gaseous substances released from the hydrothermal system is used to generate electricity to power the entire cultivation system, but quantification and optimization of the process can be done to get the exact figures for future reference and research.
6. Several resource and environmental challenges exist for scaling up of microalgal culture. To overcome this, complete detailing about the microalgal strain and the cultivation system can be done, as knowledge about the microalgal biology and biochemistry helps us understand the possible response of the microalga to a designed cultivation system with a temperature control mechanism in it as microalgae are extremely susceptible to temperature variations in open cultivation systems.
7. With monoalgal culture being a difficult target to achieve in open raceway pond systems, cultivation of algal consortium can be practiced with an effort to maintain a functional specificity of accumulating lipids rather than a species specificity.
8. With chances of microalgal spills due to cultivation of non-native microalgae in new ecosystems (Gressel et al., 2014), mutagenesis and transgenics can be explored

to delete genes that are unnecessary in culture but obligatory in nature.
9. With different harvesting techniques being experimented with for finding out a faster and efficient technique with minimum energy expenses, bioflocculation and autoflocculation have been found to be most attractive options (Vandamme et al., 2013). So research on the chemicals inside the microalgae leading to the respective phenomenon can be carried out to improvise the process and eliminate any negativity attached to the harvesting technologies.
10. Outside blown in dust, being a major impediment to harvesting costs and a reason for light shading during microalgal cultivation, can be made to settle at the bottom of the pond through some innovative flocculating mechanisms so as to improve productivity in the cultivation systems.
11. Photosynthesis being the starting point for energy capture and dissipation, the complex interplay between spectral range, light capture efficiency and CO_2_ fixation can be considered as a crucial area of research.
12. Additionally, development of models of regulatory network in microalgae to assist in better gene and metabolic regulation for optimization of the storage of chemical energy in a particular form, for understanding the signaling mechanism in algal cells in more complex algal populations and for development of predator and pathogen resistance, can allow better biological control in large scale systems.
13. Microalgal metabolism and growth rate being inversely proportional to their cell diameters, the surface-to-volume ratio of the microalgae can be considered to be an important parameter of research while searching for high biomass yielding microalgal strains.

The scope of future research in the field of microalgal biodiesel production, does not limit itself to the few points mentioned here, but goes deep into an elaboration of the points highlighted. With some research projects in progress and few more planned for the future, an analysis of the entire scenario suggests that, today at this moment, the fundamentals are the problem. Lack of fundamental knowledge on the factors governing the variations in the entire algal biodiesel production process result in vague and inconclusive impact assessment reports.

The question of whether microalgae will be a significant contributor to biodiesel production before 2030, generally depends upon the pace of innovations. Few people with a pessimistic view, are with the belief that with the current pace of microalgal biodiesel research scaling up to large quantities by 2030 will be a difficult target to achieve. However, more worrying is the fact that the pace of innovations might be slow enough to making it an uneconomic strategy to invest in at the commercial scale as compared to other opportunities.

If companies fail to innovate, they die. But if they fail to rapidly develop cash-flowing solutions, they cannot attract capital, and they die that way too. Now, cooperative research projects, with companies collaborating with institutions to develop technologies, are an old idea. But planning a roadmap of innovations by using public funds for research and development leading to company formation is a new idea that can be proposed and pursued. The near-term stance for pervasive use of microalgal biodiesel appears dreary, but biodiesel for vocation solicitations such as in aviation may be possible in the medium term.

## Conclusion

It’s honestly extremely turbulent at this moment with a large number of innovations going on at too many fronts, just to make stable forecasts about when microalgal biodiesel will become an affordable reality. It requires scientists to take too many high-risk decisions for a faster pace of innovations. However, it is very clear that counting microalgal biodiesel out, any time before 2030, is a complete no–no for the researchers. What is important to remember here is that, microalgal biodiesel is based on a system of systems, not a single technology. Hence, with patience and perseverance, that which looks daunting today will be a successfully achieved target, couple of years after.

## Author Contributions

RP read the literature and drafted the manuscript. NM supervised and corrected the manuscript. Both authors have approved the submitted version.

## Conflict of Interest

The authors declare that the research was conducted in the absence of any commercial or financial relationships that could be construed as a potential conflict of interest.

## Abbreviations

ARRA: American Recovery and Reinvestment Act
BECCS: bioenergy for carbon capture and sequestration
BTU: British thermal unit
CO_2_: carbon dioxide
EROI: energy return on investment
FER: fossil energy ratio
GGE: gallons of gasoline equivalent
GHG: greenhouse gas
ILO: International Labor Organization
IPCC: intergovernmental panel for climate change
ITUC: International Trade Union Confederation
LCA: life-cycle assessment
LCIA: life-cycle impact assessment
MJ: mega joules
NEB: net energy balance
NER: Net energy ratio
POP: pathways out of poverty
UNEP: United Nations Environment Programme.
